# Acute myocardial infarction not attributed to coronary artery disease: A seldom initial presentation of a left ventricular myxoma

**DOI:** 10.1002/ccr3.4029

**Published:** 2021-05-04

**Authors:** Kyriakos Spiliopoulos, Zacharias A. Anyfantakis, Ilias Diminikos, Andrew Xanthopoulos, Dimitrios E. Magouliotis, John Skoularigis, Filippos Triposkiadis

**Affiliations:** ^1^ Department of Thoracic and Cardiovascular Surgery Faculty of Medicine School of Health Sciences University of Thessaly Larissa Greece; ^2^ Department of Cardiology Faculty of Medicine School of Health Sciences University of Thessaly Larissa Greece; ^3^ Department of Surgery Faculty of Medicine School of Health Sciences University of Thessaly Larissa Greece

**Keywords:** cardiac tumors, myocardial infarction, myxoma, surgical excision

## Abstract

Although myxoma represents the most frequent non‐malignant cardiac primary tumor; it is extremely rare met in the left ventricle. Clinical features of the neoplasm extend from symptomless to critical signs of either ischemia or embolism. We describe here an unusual case of a huge left ventricular myxoma in a 68‐year‐old man, presented with clinical and ECG findings of an inferior wall myocardial infarction. The patient was primarily referred to our institution for coronary angiography, which showed no coronary artery disease. Further examinations revealed a left ventricular mass as the possible source of embolization, thus the patient underwent surgery for tumor excision. The postoperative course was unremarkable. A bibliographical analysis demonstrated that those tumors are rare but treatable causes of embolic myocardial infarction, thus profound clinical intuition, proper utilization of imaging modalities, administration of anticoagulants preoperatively, as well immediate surgical removal are justified.

## Graphical abstract

Myocardial infarction due to left ventricular myxoma. High clinical suspicion to establish the diagnosis is warranted.

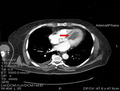

## 1 INTRODUCTION

Primary cardiac neoplasms are uncommon through all ages, with a documented incidence of up to 0.003% postmortem.^1^ They may be observed either in patients suffering from cardiovascular‐related and/or constitutional signs or coincidentally while an imaging assessment because of an irrelevant indication. Clinical presentation is mostly related to the site in the heart rather than the histological type and usually varies, including symptoms related to obstruction, embolism manifesting as myocardial infarction (MI) in the matter of coronary artery (CA) embolization, and metabolites accumulation leading to constitutional symptoms.^2^ The masses can be infiltrative, deteriorating heart function or causing valvular malfunction, thus leading subsequently to a cardiac impairment resulting in systemic and lung embolization, conduction system complications, or fateful rhythm disturbances.

We herein discuss a case of a left ventricular myxoma presented with clinical and ECG signs of an inferior wall MI. Also, we provide a brief literature review, focusing on the one on the correlation between myxomas and MI as their cardinal symptom of manifestation, and illustrating the treatment approach on the other.

The following presented patient agreed to the publication of all details including clinical images.

## 2 CASE REPORT

**FIGURE 1:**
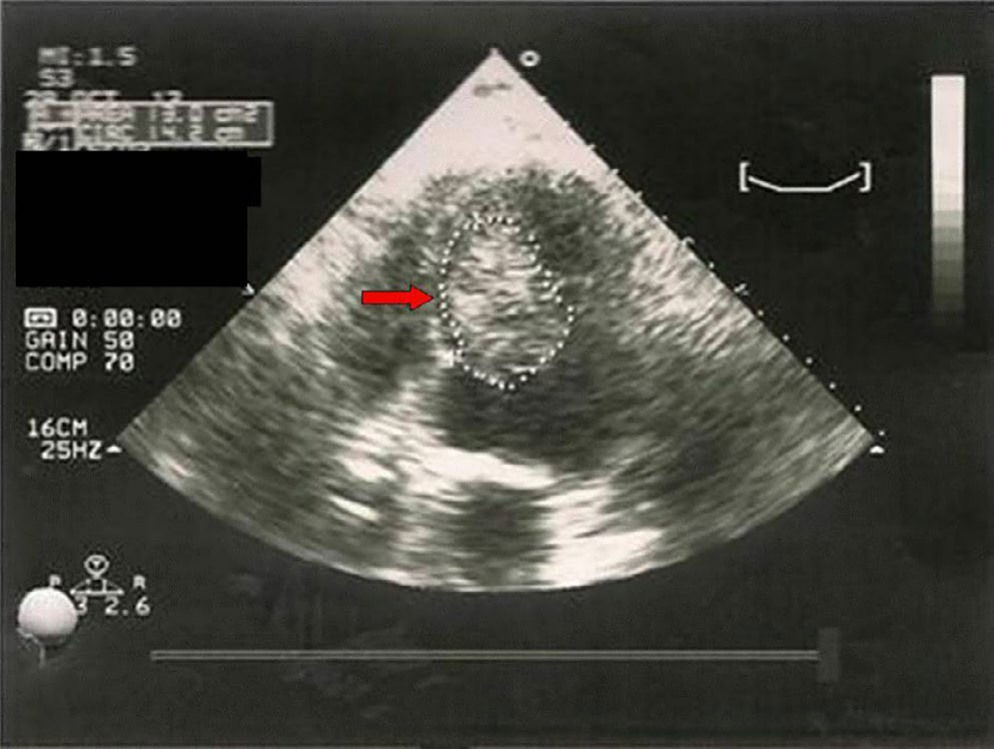
Preoperative TTE: Apical 4‐chamber view showing the tumor in the left chamber, attached to the septal wall (red arrow)

Α 68‐year‐old man was transferred from an external unit to our institution, after an inferior wall MI with ST‐segment elevation, to be subjected to a coronary angiogram. His medical history included right preauricular and left jugular area sarcomas (malignant fibrous histiocytoma) with regional relapse and focal metastasis at the right lung both treated surgically the previous year, whereas predisposing factors for cardiovascular disease were essential hypertension, hypercholesterolemia, type II diabetes, nicotine abuse as well positive family history for coronary artery disease (CAD). On physical examination at presentation, his arterial pressure was 135/85 mm Hg and normal cardiac sounds without any murmur were heard. The ECG demonstrated normal sinus rhythm and negative T waves in leads II, III, aVF, V3‐V6. A coronary angiogram, performed in our hospital, revealed no critical stenoses of the coronary arteries (CAs). On the day following coronary angiography, the patient developed multiple episodes of witnessed cardiac arrest due to polymorphic ventricular tachycardia which was treated with electric shocks. All laboratory examinations, including electrolyte levels, were normal. Transthoracic echocardiography (TTE), performed after the patient's recovery showed a left ventricular mass approximately 6.2 × 4 cm, fixed to the left ventricle septal wall (Figure 1).

**FIGURE 2:**
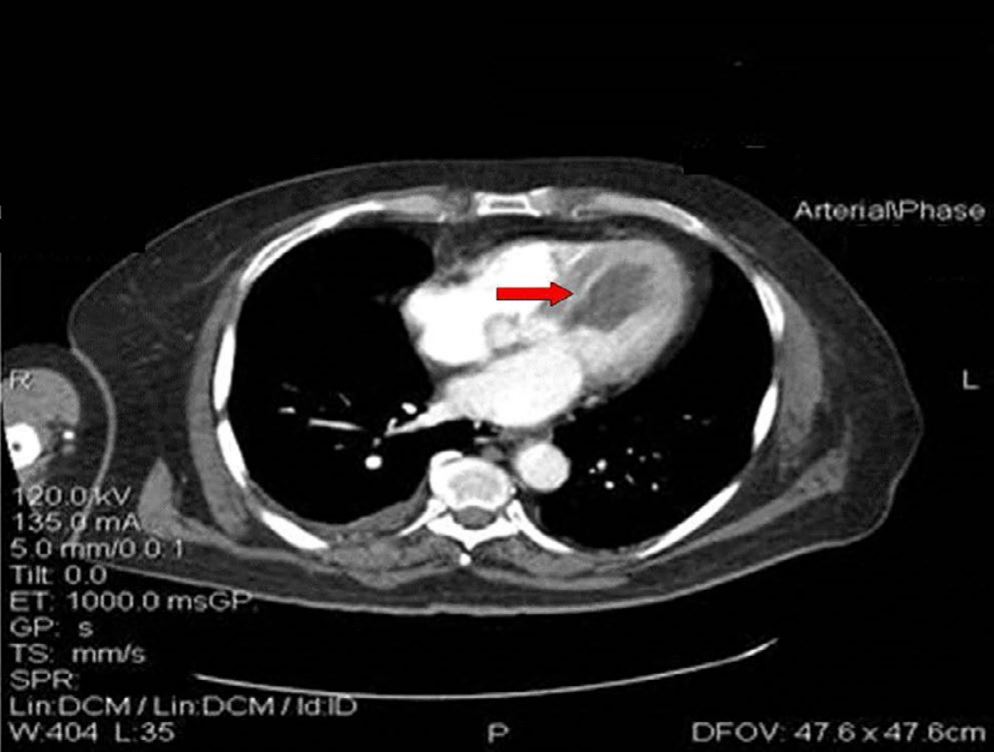
CT‐scan of the thorax: Presence of a tumor in the left chamber (red arrow)

To exclude any metastatic involvement of the heart, a computed tomography (CT) of the chest, abdomen, and pelvis was carried out demonstrating a mass within the left ventricle, extending in diameter of approximately 6.7 cm without any other significant findings (Figure 2).

**FIGURE 3:**
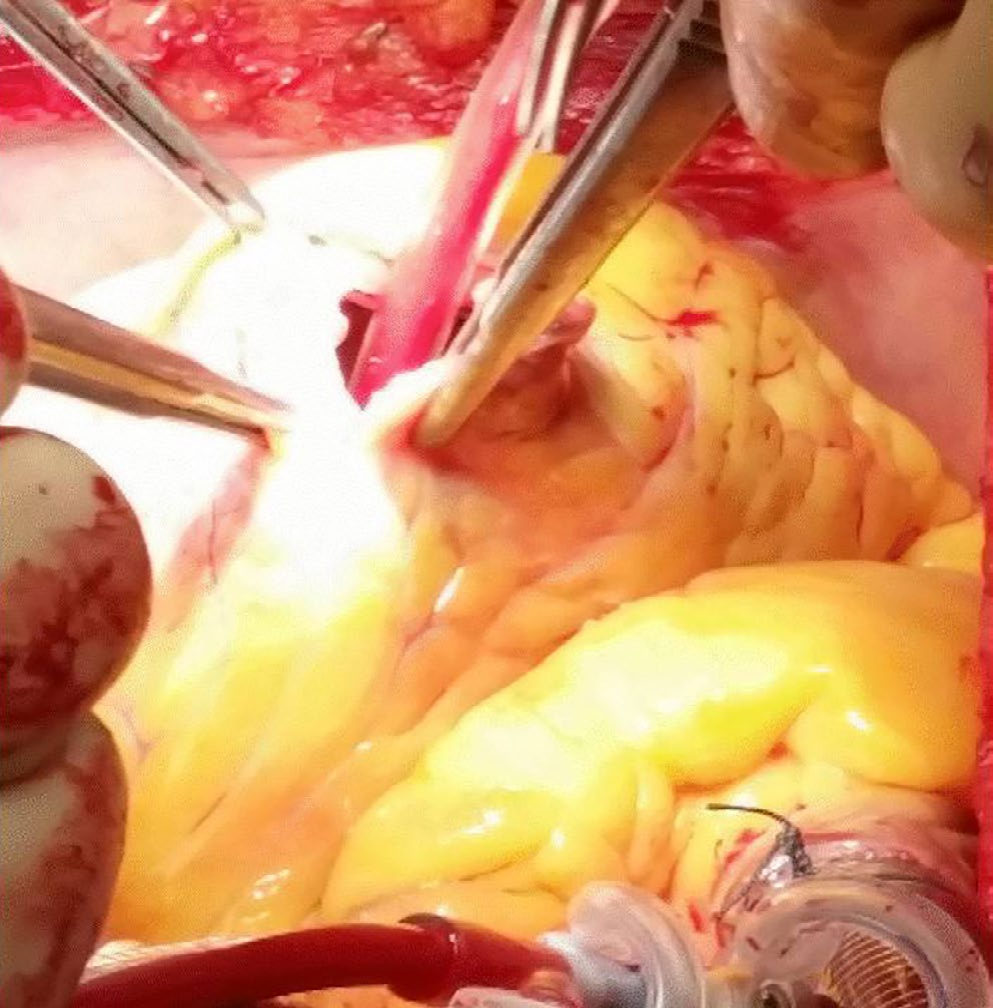
Left ventricle incision above the level of the apex with tumor resection

The patient was subsequently scheduled for transoesophageal echocardiographic study, but he developed multiple episodes of polymorphic ventricular tachycardia, uncontrolled by transvenous pacing and medical therapy, including intravenous administration of β‐blockers. Due to his critical condition, he was urgently referred for surgical excision of the mass. Through median sternotomy, ascending aorta and bicaval vein cannulation was performed. Under cardiopulmonary bypass (CPB) and cardioplegic arrest by antegrade infusion of cardioplegic solution, the left ventricle was opened over a length of 4 cm above the level of the apex parallel to the left anterior descending artery and total excision of a pedunculated tumor mass of a maximum size of 6.5 cm attached to the septal wall was performed (Figure 3). The tumor's stalk was easily shaved from the endocardium. Following, we inspected thoroughly the endocardium of the left ventricle and the mitral valve to confirm the entire tumor resection. The ventriculotomy was closed with 2 layers of continuous 3/0 polypropylene running suture supported by 2 Polytetrafluoroethylene (PTFE) strips. The patient was smoothly weaned from CPB with low dose inotropic support. Subsequent histologic examination confirmed the suspicion of myxoma. The patient did well after the procedure with an unremarkable postoperative course; therefore, he was discharged on the fifth postoperative day. He was followed up at our outpatient clinic for 18 months after surgery presenting normal TTEs without any signs of recurrence and was advised to continue semi‐annual echocardiographic controls by his cardiologist for up to 4 years postoperatively.

## 3 DISCUSSION

The majority (ca 90%) of the in general, uncommon primary cardiac neoplasms are nonmalignant,^3^ with myxoma constituting the predominant type ranging from 30%‐50%.^1^ Its annual incidence is 0.5/1 000 000 population and affects predominantly adults 30‐60 years of age, although it involves all age groups ranging from 1‐83 years. Females seem to be predisposed, as far as 65% of the tumors occur in women.^4^ Despite their sporadic development, up to 7% appears hereditary, inherited as an autosomal dominant condition accompanied by endocrine tumors and cutaneous hyperpigmentation, constituting a syndrome known as the Carney complex.^2^ They seem to occur in a multiple fashion in younger patients and atypical locations like the skin and breast. Additionally, after removal, they present an increased recurrence rate.^5^

Concerning the cardiac site of presentation, myxomas most frequently develop in the left atrium accounting of 75%,^2^ 18% appear in the right atrium, whereas a smaller number, in a total of 6%, is uniformly (3% each) distributed among the two ventricular chambers. Only an extremely low percentage of <1% affects the valves.^6^

Myxomas are typically located close to the interatrial septum at the edge of the fossa ovalis, range in diameter from 1‐15 cm, and appear polypoid with an even or slightly lobulated surface, often pedunculated with a thin pedicel. While polypoid tumors appear solid, hardly sustaining unprovoked fragmentation, villous or papillary masses are characterized by fine fragile extensions carrying a high risk of embolization.^1^ Furthermore, myxomas often contain cysts and necrotic or hemorrhagic regions. Calcification has been observed in some cases, but it is in general rare.^2^

Typical clinical symptoms of patients with myxoma include embolic, obstructive heart, and systemic/ constitutional complications.^7^ In detail, obstructive heart signs consist of vertigo, shortness of breath, tussis, lung edema, and congestive cardiac insufficiency. Regarding embolism, 30%‐40% of the cases experience embolic episodes, which practically may affect any organ.^2^ Concerning constitutional and nonembolic systemic symptoms, they occur in approximately 20% of the patients involving pyrexia, loss of weight debility, exhaustion, myalgia, arthralgia, and Raynaud's syndrome.^7^ Noteworthy, although systemic embolism is frequently observed in myxomas located in the left atrium, CA‐embolization as a tumor‐manifestation is extremely rare accounting only for 0.06%.^8^ Potential explanations for this finding may be either the coronary ostial right‐angled setting with respect to the aortic blood flow as well the ostial shielding through systolic valvular leaflet opening.^9^ Panos et al in their study evaluating 26 myxoma patients with acute MI, documented, through cardiac catheterization, embolization rates concerning the right, left anterior descending (LAD) and circumflex CA of 47.6%, 19%, and 9.5% respectively. Furthermore, 23.8% of the cases showed no CA abnormality.^10^

However, the real incidence of CA embolisms may be higher, due to a potential incomplete detection of all patients suffering from MI or sudden cardiac death.^11^ In this context, abnormal, but non‐specific, rhythm disturbances like atrial flutter/fibrillation, bundle branch block (right or left) are identified in 20%‐40% of myxoma patients, while tachyarrhythmias are observed in about 24%‐25.7% of the cases.^2^

Gold standard management for symptomatic patients is immediate surgical en bloc tumor removal with clear margins, when technically achievable. Early (30 days) mortality in most series ranges from 0‐7.5%,^6^ while the total recurrence risk after removal approaches 13%,^2^ being significantly higher in hereditary compared to sporadic tumors accounting for 22% and 3%, respectively. The recurrence‐rate rises linearly for the first 4 years postsurgery, after which the risk declines. This observation justifies the recommended semi‐annual surveillance echocardiographic follow‐up for 4 years following surgery.^2^

Regarding the surgical technique several access routes for left ventricular myxoma have been proposed including transmitral through the left atrium, transaortic through the ascending aorta, with video assistance, transseptal through the right atrium, and through a small longitudinal left ventriculotomy. In general, the selection of the approach depends mainly on tumor's location in the ventricle. For tumor masses adhered close to the left ventricular outflow tract, a transaortic approach seems more reasonable. For “deeper” localized tumors a ventriculotomy provides in our eyes better visibility and secures the mitral valve and subvalvular apparatus. On the other hand, drawbacks of this access route are a potential deterioration of the LV‐function and generation of ventricular arrhythmia.

Further general insights of the surgical technique include the meticulous treatment of the cardiac structures and tumor during its removal in order to avoid fragmentation and embolic complications, as well an extended, with clear margin resection of the tumor‐stalk and base aiming to minimize the recurrence rate.

Our patient is a rare‐interesting case of primary cardiac benign tumor, whose atypical clinical and ECG findings were those of an inferior wall MI. Moreover, the newly diagnosed cardiac tumor, despite his past medical history of recurrent extracardiac sarcomas, was not a metastatic one as it would be anticipated, but a benign primary lesion as confirmed by the histological examination. Furthermore as described before, the left ventricle as cardiac presentation‐site of myxomas, as well CA‐embolization as the first clinical sign, are extremely rare.

In addition, the differential diagnosis in the presented case includes thrombus formation in LV in the context of paraneoplastic syndrome due to the past medical history. However, as far as pedunculated globular thrombi attached to the endocardium by a very narrow stalk and moving freely within the LV are accompanied by a 60%‐80% embolization risk,^12^ patients with newly diagnosed mass suffering embolism should be treated surgically as early as possible.

Regarding the diagnostic tools, transthoracic (TT) and transesophageal echocardiography (TTE) present a high sensitivity for myxoma diagnosis of 95 and up to 100%, respectively. However, in some patients thrombi, or other masses may be misdiagnosed as myxoma.^13^ In cases having poor transthoracic echocardiographic window, TEE provides higher imaging quality. Furthermore, it offers valuable views for surgical resection regarding tumor size, location, mobility, and attachment.^13^ In terms of distinction between thrombi, benign and malignant tumors, CT or MRI provide more reliable diagnostic tools.^14^ Although CT and or MRI are not the first‐line methods for myxoma diagnosis, these technologies are in the last years definitely more and more applied. This may be explained on the one by the higher sensitivity of these techniques in revealing intracardiac lesions, and on the other by the general overuse of various diagnostic instruments.^14^

Furthermore, another interesting aspect concerns the development of life‐threatening ventricular tachycardia, which may be attributed not only to embolism but as well to abnormal activation of cardiac mechanoreceptors. Two different receptor types, predominantly found in the chamber‐wall, are distinguished. The first type is supplied by C‐fibers (unmyelinated afferent vagal nerves) responding to heart volume changes. The other receptor type is served through un‐ and myelinated fibers, moving to the spinal cord via sympathetic nerves.^15^ The two mechanoreceptors types react under standard circumstances to alterations in pre‐, afterload, and heart contractility. However, clinical as well laboratory studies showed that synchronous provocation of the two afferent fibers (sympathetic and vagal), like in case of mechanical stimulation, can augment sympathetic as well vagal afferent cardiac activity, rather than the typical reciprocity among both systems.^15^ This simultaneous activation results in increased susceptibility to fatal ventricular rhythm disturbances by reducing the fibrillation threshold.^16^ Especially, an abrupt increase in sympathetic activity has been described as a potential cause for CA spasm on the underlying heart electrical vulnerability.^17^

Although atherothrombosis represents the main cause of the acute coronary syndrome, the entity occurs as well in cases with CAs diagnosed without stenoses in cardiac catheterization. The complication of MI despite a negative angiogram has been reported for more than 30 years; however, it remains a demanding condition for physicians because of the uncertain pathophysiology, prognosis and subsequent treatment.^18^ The total incidence rate of this onset extends from 1%‐12% affecting more common women and patients of younger age.^19^ Differential diagnosis includes among others coronary microvascular disease (cardiac X‐syndrome) and imbalance between oxygen demand and supply, while in younger patients with nicotine or cocaine abuse the possibility of a CA spasm has to bear in mind.^19^

## 4 CONCLUSION

Left ventricular myxoma represents a rare entity with a quite variable clinical presentation. It includes congestive heart failure, constitutional symptoms, arrhythmias, and systemic embolization, apparently manifesting as myocardial infarction in case of CA embolism. Consequently, special attention should be paid by health providers in their everyday clinical practice, and the presence of a cardiac tumor in cases admitted with acute MI should be suspected and excluded. The establishment of a diagnosis of heart masses depends strongly on the appropriate utilization of various imaging modalities like cardiac‐TTE, CT, as well magnetic resonance. Surgical mass excision remains the treatment of choice, thus early referral to experienced centers should be encouraged, as it improves prognosis and quality of life.

## CONFLICT OF INTEREST

None declared.

## AUTHORS CONTRIBUTIONS

KS and ZAA: served as the main authors, contributed equally. ZAA, ID, KS, AX: saw the treatment of the patients, wrote the manuscript. DEM, JS, FT: drafted and revised the manuscript. All authors approved the published version.

## Data Availability

The data that support the findings of this study are available from the corresponding author upon reasonable request.
